# Improvement of High-Temperature Stability of Al_2_O_3_/Pt/ZnO/Al_2_O_3_ Film Electrode for SAW Devices by Using Al_2_O_3_ Barrier Layer

**DOI:** 10.3390/ma10121377

**Published:** 2017-12-01

**Authors:** Xingpeng Liu, Bin Peng, Wanli Zhang, Jun Zhu, Xingzhao Liu, Meng Wei

**Affiliations:** State Key Laboratory of Electronic Thin Films and Integrated Devices, University of Electronic Science and Technology of China, Chengdu 611731, China; tadyliu@outlook.com (X.L.); wlzhang@uestc.edu.cn (W.Z.); junzhu@uestc.edu.cn (J.Z.); xzliu@uestc.edu.cn (X.L.); wm2lzx1314@hotmail.com (M.W.)

**Keywords:** high temperature electrode, SAW sensor, electrical conductivity, langasite, Al_2_O_3_ barrier layer

## Abstract

In order to develop film electrodes for the surface acoustic wave (SAW) devices operating in harsh high-temperature environments, novel Al_2_O_3_/Pt/ZnO/Al_2_O_3_ multilayered film electrodes were prepared by laser molecular beam epitaxy (LMBE) at 150 °C. The first Al_2_O_3_ layer was used as a barrier layer to prevent the diffusion of Ga, La, and Si atoms from the La_3_Ga_5_SiO_14_ (LGS) substrate to the film electrode and thus improved the crystalline quality of ZnO and Pt films. It was found that the resistance of the Al_2_O_3_/Pt/ZnO/Al_2_O_3_ electrode did not vary up to a temperature of 1150 °C, suggesting a high reliability of electrode under harsh high-temperature environments. The mechanism of the stable resistance of the Al_2_O_3_/Pt/ZnO/Al_2_O_3_ film electrodes at high temperature was investigated by analyzing its microstructure. The proposed Al_2_O_3_/Pt/ZnO/Al_2_O_3_ film electrode has great potential for application in high-temperature SAW devices.

## 1. Introduction

There has been a long-standing research interest in surface acoustic wave (SAW) sensors for their wide applications in recent years [1,2,3,4]. Meanwhile, with the progress of science and technology, SAW sensors that operate stably in high temperatures are in high demand [5]. As is widely known, the major challenge in fabricating SAW sensors for operating at high temperatures is to prepare film electrodes which can work stably at high temperatures. The main existing problem is that all the film electrodes undergo rapid agglomeration, recrystallization, and atom diffusion at temperatures higher than 700 °C, resulting in the destruction of film electrodes and the failure of SAW sensors. Until now, great efforts have been made to investigate the working of film electrodes at high temperatures. Moulzolf [6,7] co-deposited Pt/Rh (10%) as film electrodes and used HfO_2_ as a passivation coating layer to hinder the agglomeration. Taguetta [8] used Ir-Rh alloy electrodes of different compositions and found that Ir-based film electrodes can withstand temperatures as high as 800 °C. Rane [9,10] used tungsten film electrodes to prevent the diffusion of Ga and O atoms from the substrate and obtained stable sensors up to 800 °C. Pereira da Cunha [11] investigated a Pt-Ni/Pt-Zr electrode containing an Al_2_O_3_ interfacial layer that was used to reduce the degradation of the electrode layer by inhibiting the interdiffusion and interfacial reactions. In our previous work [12], we prepared a AlN/Pt/ZnO multilayered electrode on Langasite (LGS, La_3_Ga_5_SiO_14_) substrate and found that the ZnO buffer layer improved the crystalline quality of Pt by inhibiting the recrystallization of Pt film at 1000 °C, showing that the crystalline quality is an important factor in determining the stability of film electrodes at high temperature. However, the abovementioned AlN/Pt/ZnO film electrode was unable to work steadily above 1100 °C because both agglomeration and the interdiffusion (of the Ga, Si, and La atoms) were difficult to control at such high temperatures.

According to previous reports, we can find that the agglomeration in metal electrodes and the interdiffusion always occur at high temperatures. It is therefore necessary to find a barrier layer to prevent the interdiffusion from substrate to the film electrode. In this work, we inserted an Al_2_O_3_ film as a barrier layer between LGS substrate and ZnO buffer layer to prevent the interfacial diffusion. The Al_2_O_3_/Pt/ZnO/Al_2_O_3_ electrode was then fabricated on LGS to explore its characteristic, from room temperature to 1200 °C.

## 2. Results and Discussion

**Figure 1:**
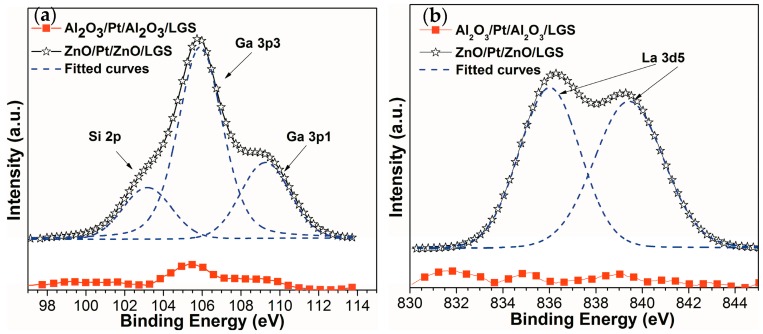
XPS (X-ray photoelectron spectroscopy) spectra of (**a**) Ga 3p, Si 2p and (**b**) La 3d5 for ZnO/Pt/ZnO/LGS and Al_2_O_3_/Pt/Al_2_O_3_/LGS samples after annealing at 1000 °C for 1 h.

Figure 1 shows the X-ray photoelectron spectroscopy (XPS) spectra of ZnO(160 nm)/Pt(30 nm)/ZnO(60 nm)/LGS and Al_2_O_3_(160 nm)/Pt(30 nm)/Al_2_O_3_(60 nm)/LGS samples after annealing at 1000 °C for 1 h. In Figure 1a, the Ga 3p and Si 2p peaks of the two samples after annealing at 1000 °C are presented. It can be seen that the intensities of the Ga 3p and Si 2p peaks of Al_2_O_3_/Pt/Al_2_O_3_/LGS sample are much lower than those of ZnO/Pt/ZnO/LGS sample. Similarly, from Figure 1b, we can see that the La 3d5 peaks of Al_2_O_3_/Pt/Al_2_O_3_/LGS sample are so low that it can barely be observed. On the other hand, the XPS spectrum of ZnO/Pt/ZnO/LGS sample shows obvious La 3d5 peaks. These results reveal the greater presence of Ga, Si, and La atoms at the surface of ZnO/Pt/ZnO/LGS sample compared to Al_2_O_3_/Pt/Al_2_O_3_/LGS sample. Therefore, it can be concluded that the Al_2_O_3_ layer can prevent the diffusion of the Ga, Si, and La atoms from the LGS substrate to film electrode and the ZnO layer cannot prevent the atoms’ diffusion. The reduction of the diffusion in Al_2_O_3_/Pt/Al_2_O_3_/LGS sample was attributed to the higher chemical stability of Al_2_O_3_ [13,14,15]. Until now, the Al_2_O_3_/Pt-Ni/Al_2_O_3_ film electrode [11] has demonstrated that the Al_2_O_3_ film can be used as a suitable diffusion barrier layer for LGS substrate. It was the reason why we focus on the film electrode by using Al_2_O_3_ barrier layer next.

**Figure 2:**
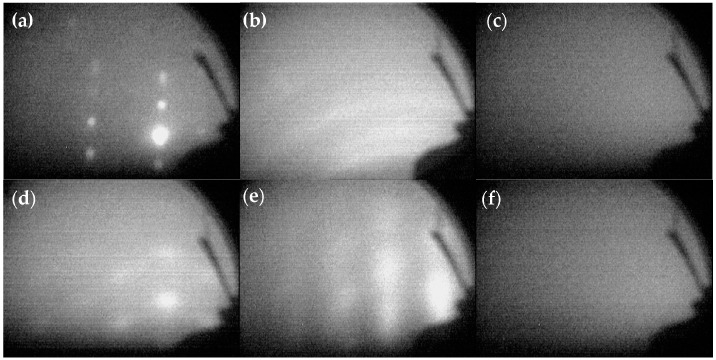
RHEED (reflected high energy electron diffraction) patterns of (**a**) a LGS substrate, (**b**) ZnO directly deposited on LGS. RHEED patterns have also been obtained for (**c**) an Al_2_O_3_ barrier layer, (**d**) ZnO, (**e**) Pt, and (**f**) the Al_2_O_3_ protective layer during the process of depositing the Al_2_O_3_/Pt/ZnO/Al_2_O_3_ electrode.

The reflected high energy electron diffraction (RHEED) patterns obtained during the deposition of Al_2_O_3_/Pt/ZnO/Al_2_O_3_ are shown in Figure 2. Figure 2a shows the bright diffraction spots of LGS that indicate that its surface was cleaned well. Figure 2b shows the RHEED pattern of ZnO directly deposited on LGS at 150 °C. The obvious diffraction rings indicate that the ZnO crystals were uniaxially textured, with the preferred orientation being out-of-plane. It shows that the ZnO deposited on LGS directly did not have sufficient crystalline quality. No diffraction spots were observed in Figure 2c, suggesting that the Al_2_O_3_ barrier layer either was not preferentially oriented or remained amorphous due to a low deposition temperature and the complete mismatch in the lattice structures of Al_2_O_3_ and LGS. However, as shown in Figure 2d, the RHEED pattern of the ZnO film deposited on an Al_2_O_3_ barrier layer shows bright diffraction spots that indicate the ZnO film was biaxially textured, with both the out-of-plane and in-plane preferred orientations. Thus, the deposition of ZnO on an Al_2_O_3_ barrier layer resulted in a better crystalline quality compared to that for a ZnO film deposited on LGS directly. In this study, we deposited 10 nm of ZnO and the ZnO buffer layer was kept so thin to reduce the influence of their mass effects on the properties of SAW devices. The Pt film deposited on the ZnO buffer layer also revealed bright diffraction spots, as shown in Figure 2e, indicating the good crystalline quality of the as-deposited Pt. Finally, the Al_2_O_3_ deposited as a protective layer was amorphous, as can be inferred from the absence of diffraction spots in Figure 2f.

**Figure 3:**
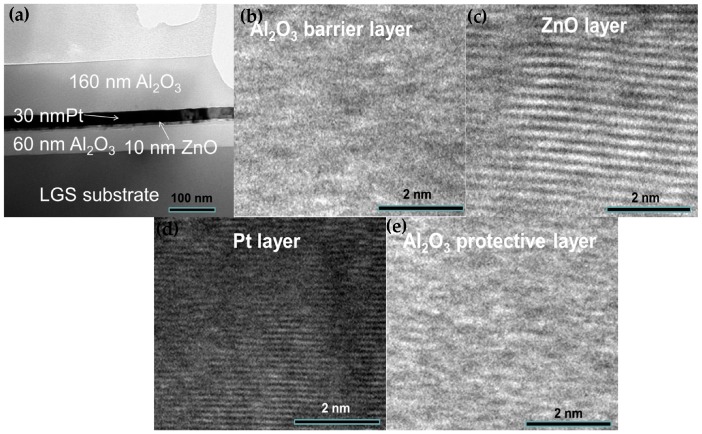
(**a**) The cross-sectional TEM (transmission electron microscope) image of Al_2_O_3_/Pt/ZnO/Al_2_O_3_/LGS sample. The enlarged HR-TEM (high resolution- transmission electron microscope) images from the regions of (**b**) Al_2_O_3_ barrier layer; (**c**) ZnO buffer layer; (**d**) Pt film; and (**e**) Al_2_O_3_ protective layer.

Figure 3a shows the cross-sectional TEM (transmission electron microscope) image of Al_2_O_3_/Pt/ZnO/Al_2_O_3_/LGS heterostructure. We can see the clear interfaces. Figure 3b–e show the enlarged cross-sectional high resolution TEM image of Al_2_O_3_ barrier layer, ZnO buffer layer, Pt layer, and Al_2_O_3_ protective layer, respectively. From these figures, we can determine that the Al_2_O_3_ layers are both in amorphous states and the ZnO, Pt films have preferred orientations. These results are consistent with the RHEED patterns as shown in Figure 2.

**Figure 4:**
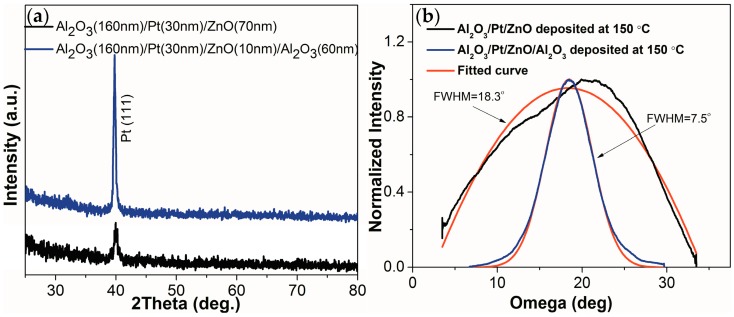
(**a**) θ–2θ XRD (X-ray diffraction) scans of the Al_2_O_3_/Pt/ZnO/Al_2_O_3_/LGS and Al_2_O_3_/Pt/ZnO/LGS samples; (**b**) Omega XRD scans of the Al_2_O_3_/Pt/ZnO/Al_2_O_3_/LGS and Al_2_O_3_/Pt/ZnO/LGS samples.

Figure 4 shows the θ–2θ and omega XRD (X-ray diffraction) scans of the Al_2_O_3_/Pt/ZnO/LGS and Al_2_O_3_/Pt/ZnO/Al_2_O_3_/LGS samples. From Figure 4a, we can see that only the Pt (111) peaks appear in these two samples. No ZnO peaks can be observed in these patterns because this this ZnO film was too thin to be detected [12]. The intensity of the Pt (111) peak is much lower for Al_2_O_3_/Pt/ZnO/LGS than Al_2_O_3_/Pt/ZnO/Al_2_O_3_/LGS. The full width at half maximum (FWHM) of these two peaks are shown in Figure 4b, and were determined to be 7.5° and 18.3° for Al_2_O_3_/Pt/ZnO/Al_2_O_3_/LGS and Al_2_O_3_/Pt/ZnO/LGS respectively. These results show that the crystalline quality of the Pt (111) layer was greatly improved by inserting the Al_2_O_3_ barrier layer. The large FWHM of the Pt peak of Al_2_O_3_/Pt/ZnO/LGS is due to the low temperature of deposition of the ZnO buffer layer, which results in the poor crystalline qualities of both ZnO and Pt. The crystalline qualities these films are typically improved by increasing the deposition temperature to 600 °C [12]. However, we have found that by inserting an Al_2_O_3_ barrier layer we can improve the crystalline quality of the ZnO buffer layer deposited at low temperatures; this was possible because of their similar crystal structures [16,17]. Thus, a Pt film of good crystalline quality could be deposited on an improved ZnO buffer layer at relatively lower temperatures. The low deposition temperature of 150 °C is very important for fabricating patterned film electrodes using lithography and the subsequent lift-off process.

**Figure 5:**
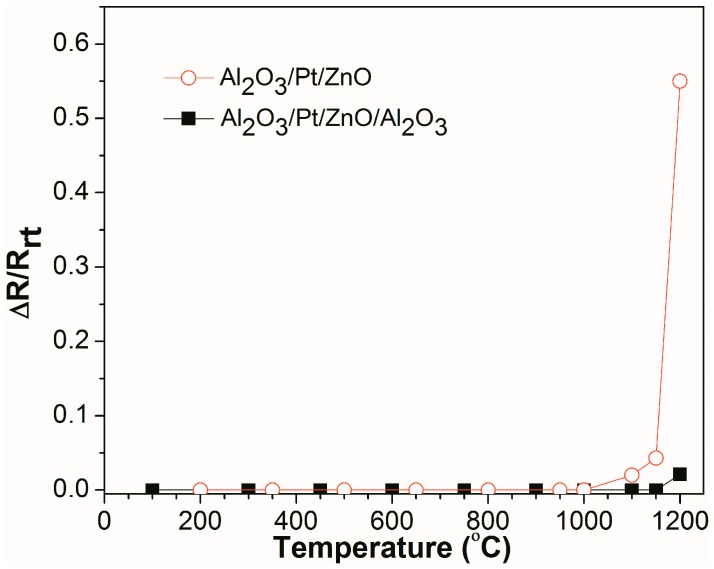
Relative resistance change for the Al_2_O_3_/Pt/ZnO/Al_2_O_3_/LGS and Al_2_O_3_/Pt/ZnO/LGS samples as a function of temperature from room temperature to 1200 °C.

Figure 5 shows the real-time, relative resistance change (ΔR/R_rt_) as a function of temperature for Al_2_O_3_/Pt/ZnO/Al_2_O_3_/LGS and Al_2_O_3_/Pt/ZnO/LGS samples, where R_rt_ is the resistance of the sample at room temperature and ΔR is the difference between the resistances at high temperature and room temperature. It can be seen that the resistance of the Al_2_O_3_/Pt/ZnO/LGS sample does not vary from room temperature to 1000 °C and increases slowly from 1000 °C to 1150 °C. From 1150 °C to 1200 °C, the resistance increases sharply. Finally, the resistance increases by 0.55 times, from room temperature to 1200 °C. For the Al_2_O_3_/Pt/ZnO/Al_2_O_3_/LGS sample, the resistance does not vary from room temperature to 1150 °C and increases by only 0.02 times from 1150 °C to 1200 °C. These results show that the stability of Al_2_O_3_/Pt/ZnO/Al_2_O_3_/LGS electrode at high temperature has been improved greatly by the Al_2_O_3_ barrier layer.

**Figure 6:**
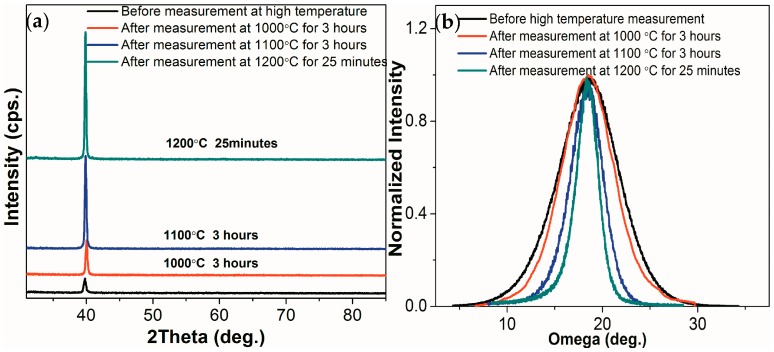
(**a**) θ–2θ scans and (**b**) omega scans of the Al_2_O_3_/Pt/ZnO/Al_2_O_3_/LGS samples before and after resistance measurement at 1000 °C, 1100 °C and 1200 °C.

Figure 6 shows the θ–2θ scans and omega scans of the Al_2_O_3_/Pt/ZnO/Al_2_O_3_/LGS samples before and after resistance measurement at 1000, 1100, and 1200 °C. As shown in Figure 6a, the intensity of Pt (111) peak doubled after the high temperature measurement at 1000 °C for 3 h. Besides, the FWHM of Pt (111) peak decreased from 7.5° to 6.8° as shown in Figure 6b after resistance measurement at 1000 °C for 3 h. Both the intensity and FWHM of the Pt (111) peaks varied by only a little after resistance measurement at 1000 °C for 3 h, showing that the recrystallization of Pt film was hindered well at 1000 °C. While, after resistance measurement at 1100 °C for 3 h and at 1200 °C for 25 min, the intensity of Pt (111) peak increased by 7 times and 10.6 times. Meanwhile, the FWHM of Pt (111) peak varied from 7.5° to 3.8° and 2.5° respectively, which indicates that the recrystallization of Pt film occurs partly at 1100 °C and the recrystallization cannot be prevented totally at 1200 °C.

**Figure 7:**
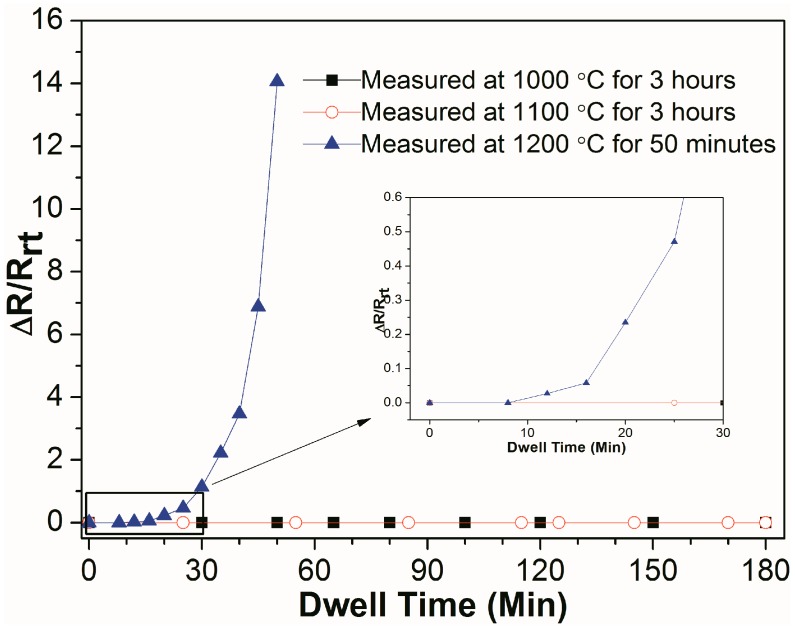
Relative resistance change of Al_2_O_3_/Pt/ZnO/Al_2_O_3_/LGS sample as a function of dwell time at 1000, 1100, and 1200 °C. The inset shows the enlarged figure of the relative resistance change curves before 30 min.

Figure 7 shows the real-time relative resistance measurement of Al_2_O_3_/Pt/ZnO/Al_2_O_3_/LGS samples at 1000 °C and 1100 °C for 3 h, and at 1200 °C for 50 min. After undergoing resistance measurement at high temperature of 1000 °C or 1100 °C for 3 h, the samples remain the same resistance all the time, indicating that the Al_2_O_3_/Pt/ZnO/Al_2_O_3_/LGS electrode can work stably at 1100 °C for 3 h. For Al_2_O_3_/Pt/ZnO/Al_2_O_3_/LGS sample measured at 1200 °C, the resistance increases slowly before 25 min and increases rapidly after 25 min. The resistance increases to infinity in 55 min. It shows that this electrode can work at 1200 °C only in a short period of time. Compared to other reported multilayer electrodes such as Pt-Rh/HfO_2_ [6] and Al_2_O_3_/Pt-Ni/Al_2_O_3_ [11], the stability of this Al_2_O_3_/Pt/ZnO/Al_2_O_3_/LGS electrode at high temperature has been improved greatly.

**Figure 8:**
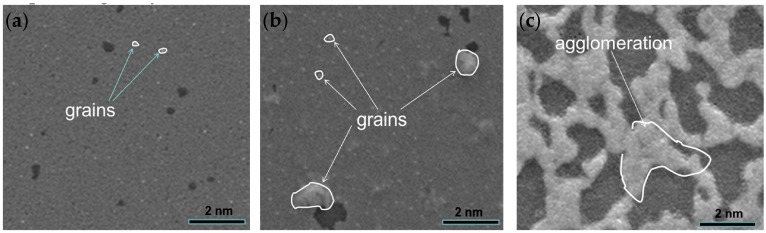
Surface topography of the Al_2_O_3_/Pt/ZnO/Al_2_O_3_/LGS samples (**a**) after resistance measurement at 1000 °C for 3 h; (**b**) after resistance measurement at 1100 °C for 3 h; and (**c**) after resistance measurement at 1200 °C for 25 min.

Figure 8 shows the surface topography of Al_2_O_3_/Pt/ZnO/Al_2_O_3_/LGS samples after high temperature resistance measurements at 1000, 1100, and 1200 °C, respectively. From Figure 8, we can see that the surface is smooth with few and small grains (marked out by curves) after resistance measurements at 1000 °C for 3 h, indicating that the film agglomerated slightly. Figure 8b shows the surface topography of Al_2_O_3_/Pt/ZnO/Al_2_O_3_/LGS sample after resistance measurement at 1100 °C for 3 h. The surface was still smooth but we can observe more and bigger grains. It shows that more severe agglomerations occurred after 1100 °C resistance measurement. However, these agglomerations are both not sufficient to increase the film resistance greatly as shown in Figure 7. Finally, it can be seen from Figure 8c that much more severe agglomeration occurred after the resistance measurement at 1200 °C for 25 min, which is similar to other film electrodes after high temperature measurements [18,19,20].

## 3. Materials and Methods

The Al_2_O_3_/Pt/ZnO/Al_2_O_3_ multilayer electrodes were grown by LMBE on LGS substrates with Euler angle of (0°, 138.5°, 116.6°) at 150 °C. A KrF (λ = 248 nm) excimer laser was operated with an energy density of about 2.5 J/cm^2^ at a frequency of 3 Hz. Highly purified Al_2_O_3_, Pt, and ZnO targets were used in this work and the distance from target to substrate was kept at 6 cm. Before depositing, the LGS substrates were ultrasonically cleaned in anhydrous alcohol for 6 min, followed by a N_2_ drying process. Then, the substrate was loaded into the deposition chamber and heated up to 150 °C. The chamber pressure was maintained at 3 × 10^−5^ Pa during growth. Finally, a 60 nm Al_2_O_3_ barrier layer, 10 nm ZnO buffer layer, and 30 nm Pt and 160 nm Al_2_O_3_ protective layer were deposited on LGS one by one. In addition, ZnO(160 nm)/Pt(30 nm)/ZnO(70 nm), Al_2_O_3_ (160 nm)/Pt(30 nm)/Al_2_O_3_(70 nm), and Al_2_O_3_(160 nm)/Pt(30 nm)/ZnO(70 nm) electrodes were also fabricated at 150 °C to study their diffusion and conductive properties at high temperature.

The growth patterns of all films were monitored by in situ RHEED diagnostic using a 20 kV electron beam. The resistances of the samples were measured from room temperature to 1200 °C in air within a tube furnace by a resistance meter (Keithley 2400, Tektronix, Cleveland, OH, USA). The heating rate was kept as 4 °C per minute. After high temperature resistance measurement, the samples were cooling down to room temperature in a natural cooling condition. Film crystal structure and texture were measured by X-ray diffraction (XRD) (D1System, Bede, Durham, UK) and transmission electron microscope (TEM) (Tecnai G2 F20, FEI, Hillsboro, OR, USA). A scanning electron microscope (SEM) (JSM-7500F, JEOL, Tokyo, Japan) was used to characterize the surface topography of the samples before and after high temperature resistance measurement. The component of each atom was characterized by the X-ray photoelectron spectroscopy (XPS) (AXIS Ultra DLD, Kratos, Manchester, UK).

## 4. Conclusions

In this work, we deposited Al_2_O_3_/Pt/ZnO/Al_2_O_3_ multilayers electrode on LGS substrate at 150 °C. It was found that the 60 nm thick Al_2_O_3_ barrier layers can prevent the diffusion of Ga, Si, and La atoms from LGS substrate to film electrode. At the same time, the depositing temperature for Pt/ZnO film with good crystalline quality can be decreased to 150 °C due to the Al_2_O_3_ barrier layer, which makes it possible to fabricate SAW devices by lithographing and lift-off process. Compared to the Al_2_O_3_/Pt/ZnO/LGS sample, the Al_2_O_3_/Pt/ZnO/Al_2_O_3_/LGS sample can bear the higher working temperature. It can work stably at 1100 °C for at least 3 h. This proposed Al_2_O_3_/Pt/ZnO/Al_2_O_3_ film electrode has great potential for applications in SAW sensors and many other sensors which would work in harsh, high-temperature environments.
